# Supplementary material for: Rapid Production and Genetic Stability of Human Mesenchymal Progenitor Cells Derived from Human Somatic Cell Nuclear Transfer-Derived Pluripotent Stem Cells
Source: Int J Mol Sci. 2021 Aug 26;22(17):9238. doi: 10.3390/ijms22179238 (PMC8431643; doi:10.3390/ijms22179238)
Supplement: Supplementary file 1 [file ijms-22-09238-s001.zip › ijms-1334771-supplementary.pdf]

Rapid production and genetic stability of human mesenchymal progenitor cells derived from human somatic cell nuclear transfer-derived pluripotent stem cells

Soo Kyung Jung [1,†], Jeoung Eun Lee [1,†], Chang Woo Lee [1], Sung Han Shim [2] and Dong Ryul Lee [1,2,*]

[1] CHA Advanced Research Institute, CHA University Bundang Medical Center, Seongnam, Gyeonggi 13488, Korea; bellotsk@hanmail.net (S.K.J.); jel43@chamc.co.kr (J.E.L.); lcwckddn@chamc.co.kr (C.W.L.)

[2] Department of Biomedical Science, CHA University, Seongnam, Gyeonggi 13488, Korea; shshim@cha.ac.kr

* Correspondence: drleedr@cha.ac.kr

† These authors contributed equally to this work.

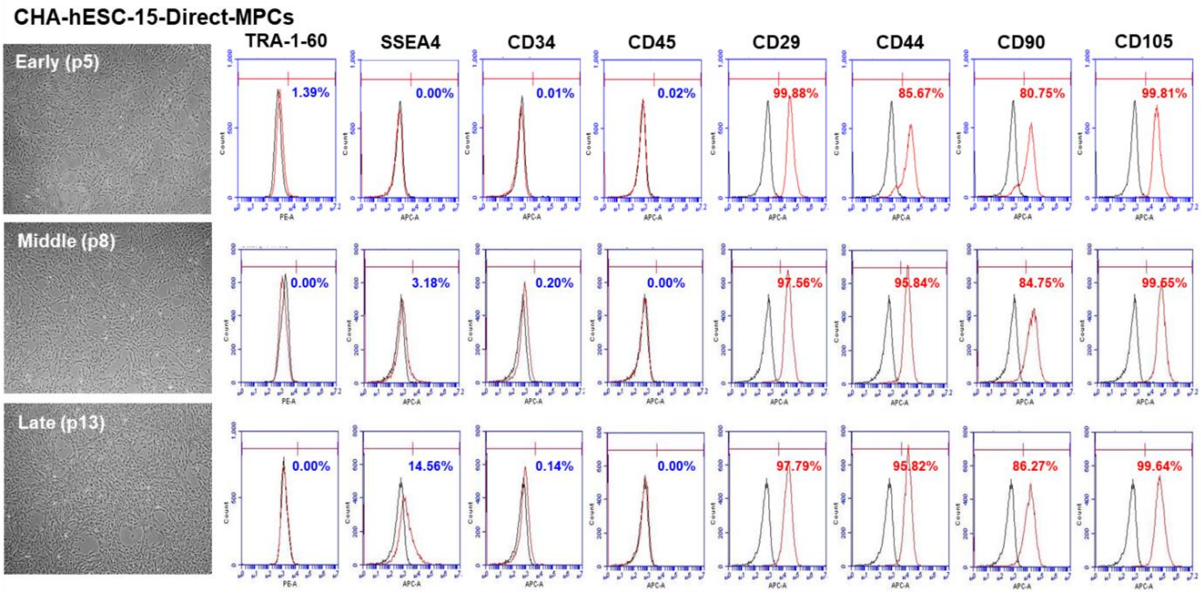

Supplementary figure S1. Representative morphology and surface antigen expression profiles of CHA-hESC-15-Direct-MPCs during subculture. Over time, the morphologies of subcultured hPSC-Direct-MPCs were similar regardless of the number of passages. Bars, 100 μm (magnification, 40×). Additionally, FACS analysis showed that the markers for MPCs (CD29, CD44, CD90 and CD105) were highly expressed, but neither the pluripotent stem cell markers (TRA-1-60 and SSEA4) nor the hematopoietic markers (CD34 and CD45) were highly expressed, regardless of the number of passages.

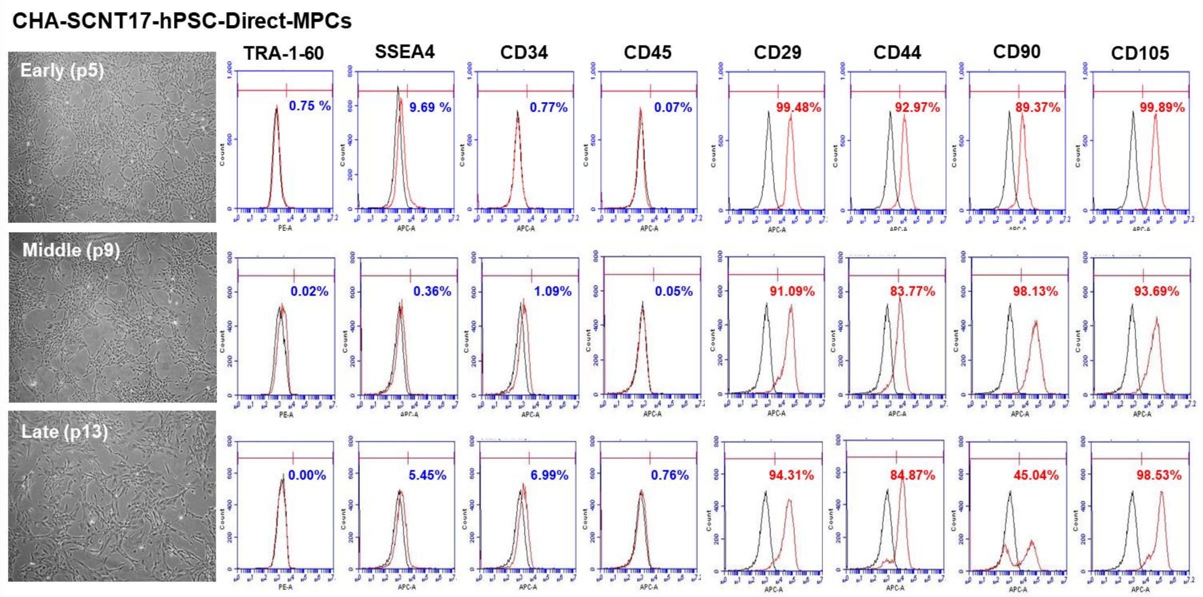

Supplementary figure S2. Representative morphology and surface antigen expression profiles of CHA-SCNT-hPSC-17-Direct-MPCs during subculture. Over time, the morphologies of subcultured hPSC-Direct-MPCs were similar regardless of the number of passages. Bars, 100 μm (magnification, 40×). Additionally, FACS analysis showed that the markers for MPCs (CD29, CD44, CD90 and CD105) were highly expressed, but neither the pluripotent stem cell markers (TRA-1-60 and SSEA4) nor the hematopoietic markers (CD34 and CD45) were highly expressed, regardless of the number of passages.

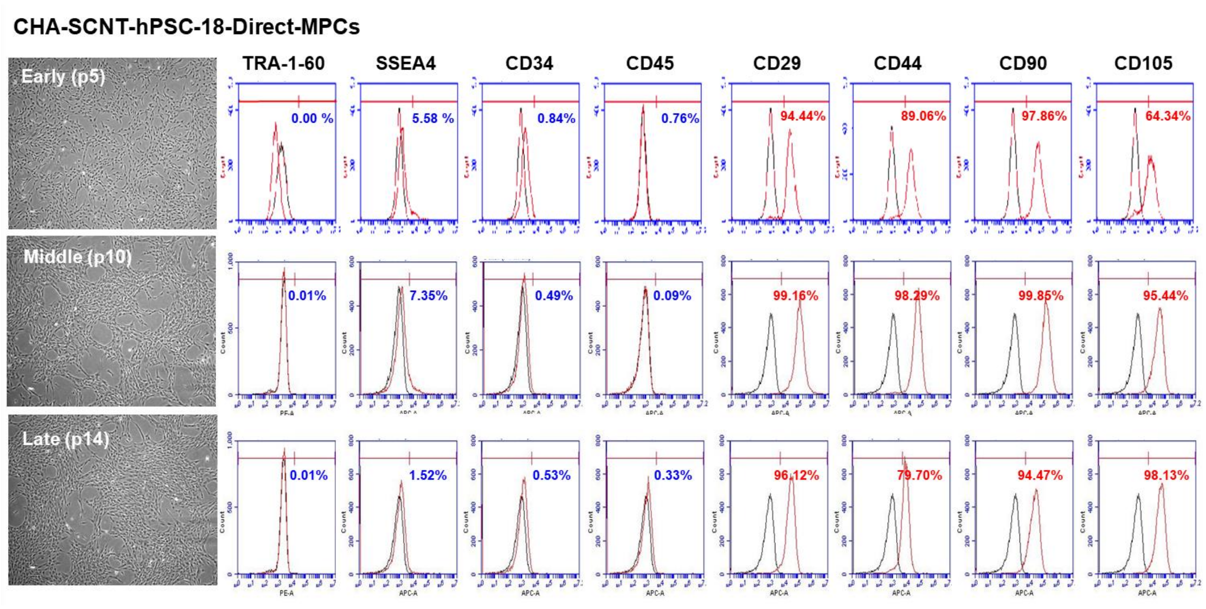

Supplementary figure S3. Representative morphology and surface antigen expression profiles of CHA-SCNT-hPSC-18-Direct-MPCs during subculture. Over time, the morphologies of subcultured hPSC-Direct-MPCs were similar regardless of the number of passages. Bars, 100 μm (magnification, 40×). Also, FACS analysis showed that the markers for MPCs (CD29, CD44, CD90 and CD105) were highly expressed, but neither the pluripotent stem cell markers (TRA-1-60 and SSEA4) nor the hematopoietic markers (CD34 and CD45) were highly expressed, regardless of the number of passages.

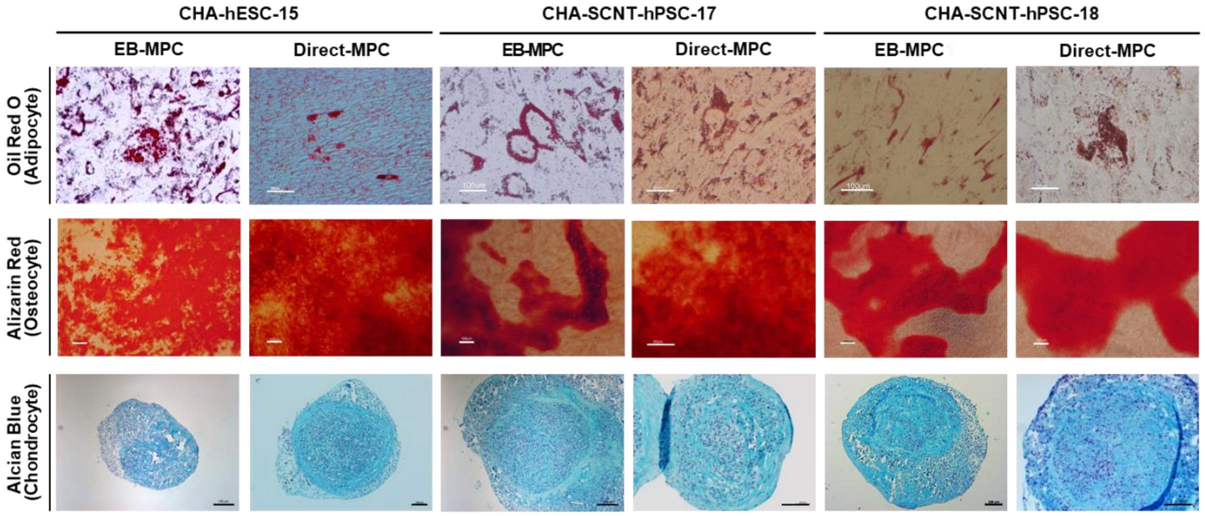

Supplementary figure S4. Differentiation ability of hPSC-derived MPCs (passages 5 to 7) into adipocytes, chondrocytes, and osteocytes. The lipid droplets in adipocytes were stained with Oil Red O. The calcium deposits at the intracellular region in osteocytes were stained with Alizarin Red. Cells that differentiated into chondrocytes were fixed and then embedded in paraffin. The sectioned samples were stained with Alcian Blue. Bar size, 100 µm.

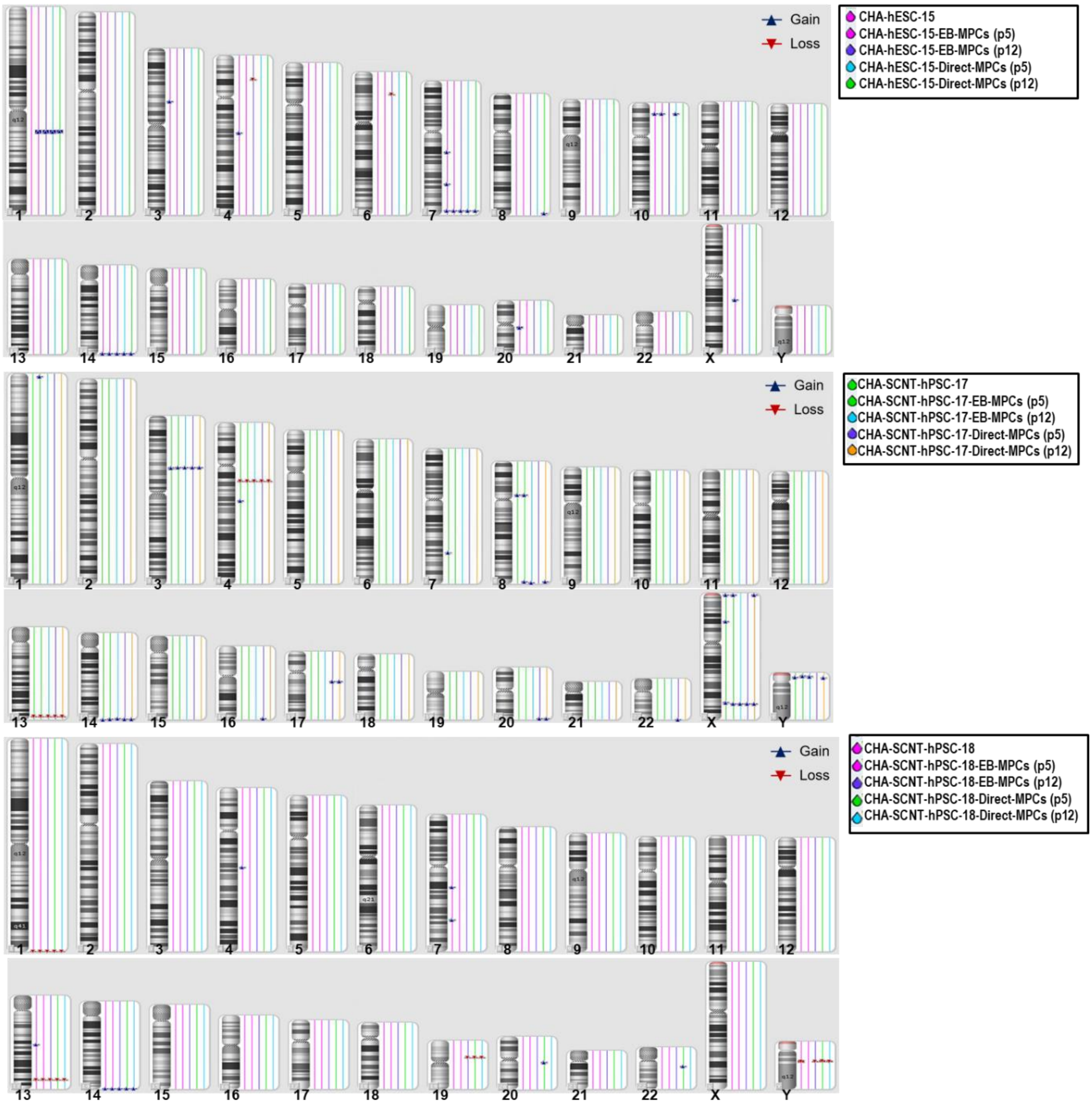

Supplementary figure S5. Ideogram illustrating the genome-wide distribution of DNA amplifications and deletions (copy number variation) on each chromosome of hPSC and hPSC-derived MPCs at early and late passages. Each line next to a chromosome represents one sample. Amplifications and deletions are mapped onto the chromosome. Each individual CNV is marked: blue triangles pointing upward indicate amplification, red triangles pointing downward indicate deletion. Variation size > 100 kbp.

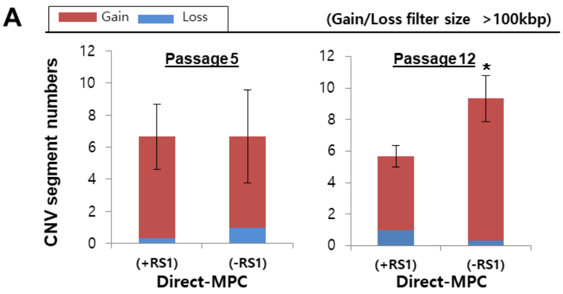

Cell lines	Passage. 5			Passage. 12		
	Gain	Loss	Total	Gain	Loss	Total
CHA-hESC-15-D(+RS1)-MPC	3	0	**3**	3	2	**5**
CHA-SCNT-hPSC-17-D(+RS1)-MPC	10	0	**10**	7	0	**7**
CHA-SCNT-hPSC-18-D(+RS1)-MPC	6	1	**7**	4	1	**5**
**Total count**	19.00	1.00	**20.00**	14.00	3.00	**17.00**
**Average**	6.33	0.33	**6.67**	4.67	1.00	**5.67**
**STDEV**	3.51	0.58	**3.51**	2.08	1.00	**1.15**
**SEM**	2.03	0.33	**2.03**	1.20	0.58	**0.67**
CHA-hESC-15-D(-RS1)-MPC	2	0	**2**	9	0	**9**
CHA-SCNT-hPSC-17-D(-RS1)-MPC	10	2	**12**	12	0	**12**
CHA-SCNT-hPSC-18-D(-RS1)-MPC	5	1	**6**	6	1	**7**
**Total count**	17.00	3.00	**20.00**	27.00	1.00	**28.00**
**Average**	5.67	1.00	**6.67**	9.00	0.33	**9.33**
**STDEV**	4.04	1.00	**5.03**	3.00	0.58	**2.52**
**SEM**	2.33	0.58	**2.91**	1.73	0.33	**1.45**
***P* value (t-test)**		**0.500**			**0.027**	

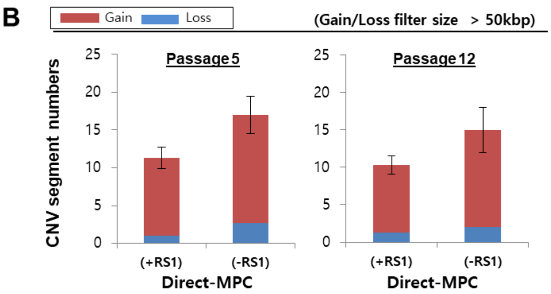

Cell lines	Passage. 5			Passage. 12		
	Gain	Loss	Total	Gain	Loss	Total
CHA-hESC-15-D(+RS1)-MPC	9	0	**9**	10	1	**11**
CHA-SCNT-hPSC-17-D(+RS1)-MPC	14	0	**14**	12	0	**12**
CHA-SCNT-hPSC-18-D(+RS1)-MPC	8	3	**11**	5	3	**8**
**Total count**	31.00	3.00	**34.00**	27.00	4.00	**31.00**
**Average**	10.33	1.00	**11.33**	9.00	1.33	**10.33**
**STDEV**	3.21	1.73	**2.52**	3.61	1.53	**2.08**
**SEM**	1.86	1.00	**1.45**	2.08	0.88	**1.20**
CHA-hESC-15-D(-RS1)-MPC	18	2	**20**	11	1	**12**
CHA-SCNT-hPSC-17-D(-RS1)-MPC	17	2	**19**	19	2	**21**
CHA-SCNT-hPSC-18-D(-RS1)-MPC	8	4	**12**	9	3	**12**
**Total count**	43.00	8.00	**51.00**	39.00	6.00	**45.00**
**Average**	14.33	2.67	**17.00**	13.00	2.00	**15.00**
**STDEV**	5.51	1.15	**4.36**	5.29	1.00	**5.20**
**SEM**	3.18	0.67	**2.52**	3.06	0.58	**3.00**
***P* value (t-test)**		**0.095**			**0.092**	

Supplementary figure S6. Array-based comparative genomic hybridization analysis of hPSC-derived Direct-MPCs after the addition of RS1. Human PSCs were used as the control cells in an analysis of array-CGH results for genomic variation. CNV results are presented as the means of MPCs derived from three cell lines: CHA-hESC-15, CHA-SCNT-hPSC-17, or CHA-SCNT-hPSC-18. The data are presented as the means ± SE. *p<0.05. (A) Variation size > 100 kbp and (B) variation size > 50 kbp.

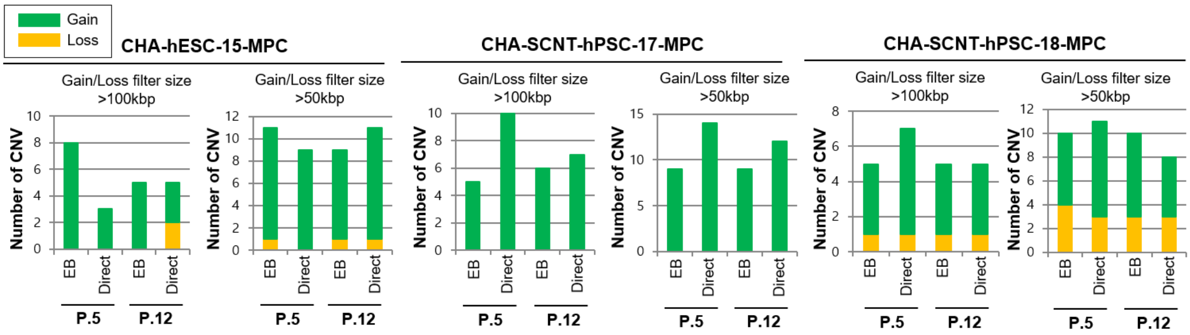

Supplementary figure S7. Copy number variation results for each hPSC-derived-MPC lines related to Figure 4.

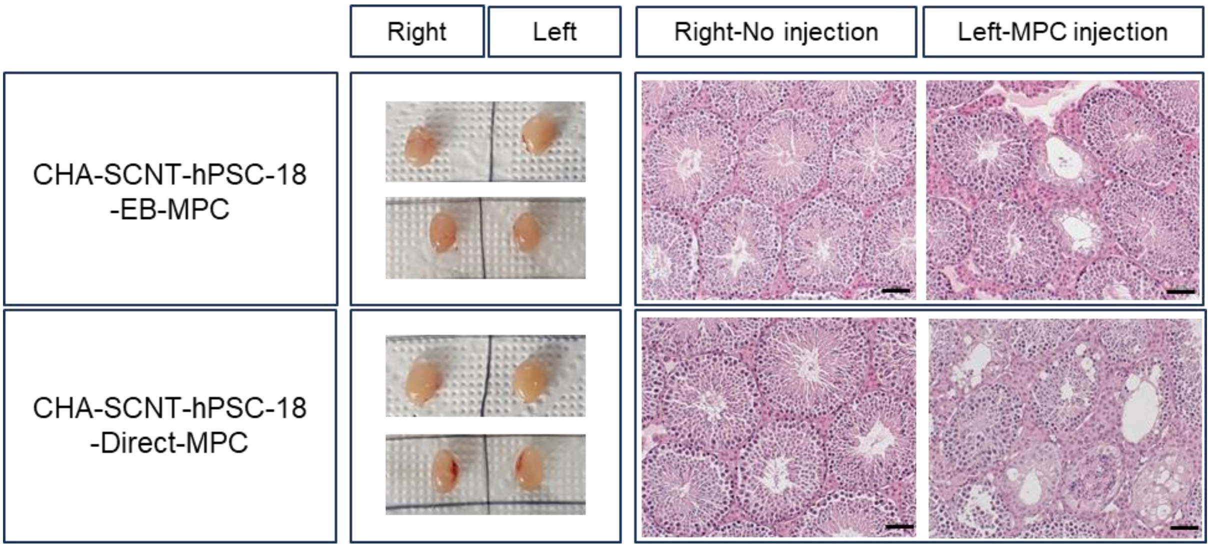

Supplementary figure S8. Nontumorigenic properties of CHA-SCNT-hPSC-18-derived MPCs. CHA-SCNT-hPSC-18-EB-MPCs and CHA-SCNT-hPSC-18-Direct-MPCs were transplanted into the left testis of immunodeficient mice. No teratomas in either the CHA-SCNT-hPSC-18-EB-MPC or CHA-SCNT-hPSC-18-Direct-MPC (p5~p6) injected mice were observed after 15 weeks. Bar size, 50 μm.

Supplementary Table S1. CNV size and related genes in hPSC-derived EB-MPCs and Direct-MPCs (variation size >100 kbp)

	Chr.	Start	End	Size(kb)	Genes in this region
CHA-hESC-15 EB *vs.* Direct-MPCs					None
CHA-SCNT-hPSC-17 EB *vs.* Direct-MPCs	1	1220665	2820376	1599711	SDF4, B3GALT6, FAM132A, SCNN1D, CPSF3L, CPTP, TAS1R3, DVL1, AURKAIP1, CCNL2, MRPL20, VWA1, ATAD3B, ATAD3A, TMEM240, MIB2, MMP23B, MMP23A, CDK11B, CDK11A, NADK, GNB1, CALML6, GABRD, PRKCZ, FAAP20, SKI, PEX10, PLCH2, PANK4, HES5, TNFRSF14
	16	88726738	88899909	173171	PIEZO1, CDT1, APRT, GALNS, TRAPPC2L, CBFA2T3
	17	36098850	36466620	367770	CCL4, TBC1D3B, CCL3L3, CCL3L1, CCL4L1, CCL4L2
	20	62315179	62663307	348128	LAMA5, RPS21, GATA5, MIR1-1, MIR133A2, SLCO4A1
	22	50148437	50291244	142807	MOV10L1, PANX2, TUBGCP6, HDAC10, MAPK12, MAPK11, PLXNB2
CHA-SCNT-hPSC-18 EB *vs.* Direct-MPCs	20	31245731	32159581	913850	DEFB118, DEFB119. DEFB121, DEFB122, DEFB123, REM1, HM13, ID1, COX4I2, BCL2L1, ABALON, TPX2, MYLK2, FOXS1, DUSP15, PDRG1, HCK
	22	22655866	22916863	260997	MIR650
